# Dual Antiplatelet Therapy Can Be Discontinued at Three Months after Implantation of Zotarolimus-Eluting Stent in Patients with Coronary Artery Disease

**DOI:** 10.1155/2013/518968

**Published:** 2013-04-04

**Authors:** Tadashi Wada, Makoto Nakahama, Hironobu Toda, Atsuyuki Watanabe, Katsushi Hashimoto, Ritsuko Terasaka, Kazufumi Nakamura, Nobuyuki Yamada, Hiroshi Ito

**Affiliations:** ^1^Department of Cardiology, Fukuyama City Hospital, 5-23-1 Zaou-chou, Fukuyama, Hiroshima 721-8511, Japan; ^2^Department of Cardiovascular Medicine, Okayama University Graduate School of Medicine, Dentistry and Pharmaceutical Sciences, 2-5-1 Shikata-cho, Kita-ku, Okayama 700-8558, Japan

## Abstract

Dual antiplatelet therapy (DAPT) after percutaneous coronary intervention increases the risk of bleeding. We studied the safety and clinical outcomes of switching from DAPT to aspirin monotherapy at 3 months after ZES implantation. We retrospectively evaluated 168 consecutive patients with coronary artery disease who had been implanted with a ZES from June 2009 through March 2010. After excluding 40 patients according to exclusion criteria such as myocardial infarction, 128 patients were divided into a 3-month DAPT group (67 patients, 88 lesions) and a 12-month conventional DAPT group (61 patients, 81 lesions). Coronary angiographic followup and clinical followup were conducted at more than 8 months and at 12 months after ZES implantation, respectively. Minor and major bleeding events, stent thrombosis (ST), and major adverse cardiac events (MACE) (death, myocardial infarction, cerebrovascular accident, target lesion revascularization, and target vessel revascularization) were evaluated. There were no statistically significant differences in the incidences of ST and MACE between the two groups. The incidence of bleeding events was significantly lower in the 3-month group than in the 12-month group (1.5% versus 11.5%, *P* < 0.05). DAPT can be safely discontinued at 3 months after ZES implantation, which reduces bleeding risk.

## 1. Introduction

DESs have reduced the incidences of in-stent restenosis and target lesion revascularization (TLR) compared to those with bare-metal stents (BMS). The 2007 Focused Update of ACC/AHA/SCAI Guidelines for Percutaneous Coronary Intervention (PCI) recommends dual antiplatelet therapy (DAPT) with aspirin and a thienopyridine drug ideally up to 12 months after DES implantation [1]. This long DAPT after DES implantation is associated with an increased risk of bleeding, for example, gastrointestinal bleeding and intracranial hemorrhage [2–11]. Major bleeding may deteriorate the quality of life of patients by deferring an endoscopic, dental, or surgical procedure [12].

Little evidence about the optimal duration of DAPT is available [4, 7]. Recent studies demonstrated that the duration of DAPT might be shortening when an endeavor zotarolimus-eluting stent (ZES, Medtronic Inc., Santa Rosa, CA) is used. ZES is a second-generation cobalt-alloy DES that has a highly biocompatible polymer permitting rapid release of the antiproliferative substance zotarolimus. ZES has been reported (1) to accelerate arterial healing compared with other DESs in animals [13–15], (2) to be safe in humans for 5 years [16], and (3) to have a low risk for development of myocardial infarction (MI) and late stent thrombosis (ST) compared with a paclitaxel-eluting stent [17]. The aim of the present study was to determine whether DAPT can be safely switched to aspirin monotherapy as early as 3 months after ZES implantation.

## 2. Materials and Methods

### 2.1. Study Population

We retrospectively studied 168 consecutive patients (239 lesions) with significant coronary stenosis who underwent PCI with ZES at our hospital from June 2009 through March 2010. As ZES has good product profiles for a short duration of DAPT and the description of ZES mentions discontinuing DAPT at 3 months, we had switched from DAPT to aspirin monotherapy at 3 months in patients who could have accepted our informed consent.

**Figure 1:**
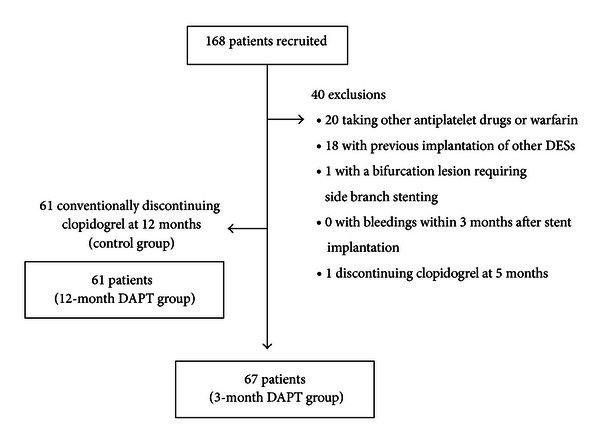
Enrollment of patients. DAPT: dual antiplatelet therapy.

In this study, patients were eligible for participation based on the following inclusion criteria (1) clinically significant coronary stenosis (diameter stenosis >70% or diameter stenosis >50% with objective evidence of myocardial ischemia), (2) *de novo *coronary restenotic lesions, and (3) restenotic coronary lesions. Exclusion criteria were (1) acute MI, (2) receiving antiplatelet agents other than aspirin and clopidogrel, (3) receiving antithrombotic drugs (e.g., warfarin), (4) expected survival of less than 1 year, (5) major bleeding within 3 months of PCI, (6) previously having undergone PCI with other DESs, (7) bifurcation lesions requiring side branch stenting, and (8) not switching from DAPT to aspirin monotherapy at 3 months or 12 months after ZES implantation. There were 168 patients who met the inclusion criteria, and 40 patients had one of the exclusion criteria. Consequently, there were 67 patients who switched from DAPT to aspirin monotherapy at 3 months and they were assigned to a 3-month DAPT group. 61 patients, who conventionally continued DAPT for 12 months, were assigned to a control group (12-month DAPT group). Figure 1 shows the enrollment of patients. The protocol of the present study was approved by the ethics committee at our hospital, and all patients provided written informed consent.

### 2.2. PCI Procedure and Protocol of Antiplatelet Therapy

If patients had not received oral antiplatelet agents, loading doses of aspirin (100–200 mg/day) and clopidogrel (300 mg/day) were given before PCI. Routine anticoagulation therapy was performed during PCI. All procedures were performed with the standard interventional techniques. Predilation, stent implantation, postdilation, and intravascular ultrasound were conducted at the discretion of the operator. Aspirin (100 mg/day) was given indefinitely after ZES implantation. Clopidogrel (75 mg/day) was discontinued at 3 months after ZES implantation and switched to aspirin monotherapy in patients who had accepted our informed consent (3-month DAPT group), while DAPT was continued for 12 months after ZES in other patients as usual (12-month DAPT group).

### 2.3. Followup and Endpoints

Patient's information was collected from medical records and by phone contact when the patient did not visit the hospital. Clinical followup was performed by medical examination on an outpatient basis or by phone at 1, 3, 8, and 12 months after ZES implantation. Drug adherence and bleeding events were assessed at the time of followup. Coronary angiographic followup was performed whenever possible in patients at more than 8 months after ZES implantation. Quantitative coronary angiography (QCA) was conducted based on coronary angiograms obtained before, immediately after, and at 8 months after ZES implantation. Reference vessel diameter (RVD), minimal lumen diameter (MLD), and lesion length were measured by experienced angiographers (N.M., K.H., A.W., and T.W.). Acute gain was calculated as the difference between post- and preprocedural MLDs. Late loss was defined as the difference between postprocedural and late MLDs. Stent restenosis was defined as a percent diameter stenosis (%DS) value of ≥50%.

The primary endpoints were minor and major bleeding events and ST. Early (≤30 days) and late (31–365 days), STs (“Definite,” “Probable,” or “Possible”) were assessed on the basis of Academic Research Consortium (ARC) criteria [18]. Definitions of bleeding events were based on the PCI CURE study [19]: major bleeding was defined as bleeding that was significantly disabling, intraocular, or requiring at least 2 units of blood. Major bleeding was subclassified as life threatening if it was fatal, if it led to a decrease in hemoglobin concentration of 50 g/L, if it caused significant hypotension requiring intravenous inotropes or surgical intervention, if it resulted in symptomatic intracranial hemorrhage, or if it necessitated transfusion of 4 or more units of blood; and minor bleeding was defined as bleeding that led to interruption of study medication.

The secondary endpoints were major adverse cardiac events (MACE) (cardiac death, MI, cerebrovascular accident (CVA), TLR, and target vessel revascularization (TVR)). MI was defined as the presence of clinical signs involving increases in creatine kinase MB and troponin T that exceeded the reference values. Periprocedural MI was excluded from this study. TLR and TVR were defined as repeat PCI or bypass graft surgery for the target lesion and vessel, respectively. 

### 2.4. Statistical Analysis

Continuous variables were expressed as mean ± standard deviation. The means between the two groups were tested by Student's *t*-test or the Mann-Whitney *U* test. Discontinuous variables and nominal variables were expressed as frequencies and percentages. Differences between the two groups were tested by the *χ* ^2^ testor Fisher's test. A value of *P* < 0.05 was considered statistically significant. JMP7 (SAS Institute, Cary, NC) was used for the analysis.

## 3. Results

### 3.1. Baseline Characteristics of Patients

Patients assessed were 168 consecutive patients (239 lesions) who underwent PCI with ZES for coronary artery disease (CAD) at our hospital from June 2009 through March 2010. Among them, 39 patients (69 lesions) were excluded because they took warfarin and an antiplatelet agent other than aspirin and clopidogrel, had previously undergone implantation of other DESs, or had bifurcation lesions requiring side branch stenting. In addition, 1 patient (1 lesion) stopped taking clopidogrel at 5 months after ZES implantation. Consequently, 67 patients (88 lesions) and 61 patients (81 lesions) were enrolled in the 3- and 12-month DAPT groups, respectively (Figure 1). Baseline characteristics of patients are shown in Table 1. Rations of males (64.8% versus 51.8%, *P* = 0.05) and smokers (50.8% versus 34.3%, *P* = 0.02) were higher in the 3-month DAPT group than in the 12-month DAPT group. However, no statistically significant difference was found between the two groups with respect to other baseline characteristics of patients. Patients in the two groups had a relatively high mean age of about 70 years and included high-risk patients for ST, that is, patients with decreased renal function and patients with decreased cardiac function caused by old MI.

### 3.2. Lesion Characteristics and Angiographic Outcomes

Lesion characteristics and coronary angiographic outcomes are shown in Table 2. At baseline, lesion length was significantly shorter in the 3-month DAPT group than in the 12-month DAPT group (13.3 ± 7.04 mm versus 15.6 ± 5.82 mm, *P* = 0.02). However, there was no statistically significant difference in the percentage of left main trunk (LMT) lesions (4.9% versus 7.4%, *P* = 0.31). At 8 months afterprocedure, no statistically significant differences were found between the 3- and 12-month DAPT groups in the following variables: TLR (9.1% versus 9.9%, *P* = 0.52), TVR (9.1% versus 9.9%, *P* = 0.52), QCA [RVD (2.38 ± 0.53 mm versus 2.64 ± 0.51 mm, *P* = 0.31), MLD (2.24 ± 0.58 mm versus 2.16 ± 0.79 mm, *P* = 0.46), %DS (19.7 ± 17.3% versus 21.4 ± 20.2%, *P* = 0.27), and late lumen loss (0.45 ± 0.52 mm versus 0.51 ± 0.47 mm, *P* = 0.51)].

### 3.3. Primary and Secondary Endpoints

**Figure 2:**
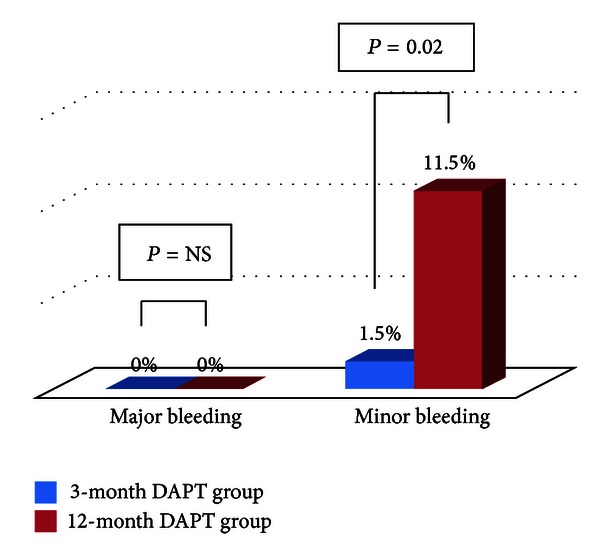
Incidences of bleeding events in the 3- and 12-month DAPT groups. DAPT: dual antiplatelet therapy; NS: not significant.

All patients could be followed up clinically (mean follow-up period: 411 ± 62 days). The incidences of bleeding events during the follow-up period are shown in Figure 2. Major bleeding was not observed in either group. The incidence of minor bleeding events was significantly lower in the 3-month DAPT group than in the 12-month DAPT group (1.5% versus 11.5%, *P* = 0.02). In the 3-month DAPT group, one patient had upper gastrointestinal bleeding at 6 months after ZES implantation. In the 12-month DAPT group, nasal bleeding that required hemostasis in the Department of Otorhinolaryngology was found in 4 patients (2, 4, 5, and 9 months after ZES implantation, resp.), lower gastrointestinal bleeding was found in 2 patients (4 and 7 months after ZES implantation, resp.), and upper gastrointestinal bleeding was found in one patient (6 months after ZES implantation). The incidences of ST in the two groups after ZES implantation are shown in Table 3. Although the 12-month DAPT group included one patient with ST, no significant difference was found between the two groups (0% versus 1.2%, *P* = 0.38).

The incidences of MACE are shown in Table 4. No significant differences were found between the 3- and 12-month DAPT groups in the following variables: MACE (11.4% versus 11.1%, *P* = 0.48); cardiac death (0% versus 1.2%, *P* = 0.38), MI (all causes) (2.3% versus 1.2%, *P* = 0.48), Q-MI (1.1% versus 1.2%, *P* = 0.64), non-Q-MI (1.1% versus 0%, *P* = 0.41), TLR (9.1% versus 9.9%, *P* = 0.52), and TVR (9.1% versus 9.9%, *P* = 0.52).

## 4. Discussion

Our study demonstrated that switching from DAPT to aspirin monotherapy at 3 months after ZES implantation was not associated with an increased incidence of ST or MACE but was associated with a reduction in the incidence of minor bleeding. To the best of our knowledge, the present study is the first study to demonstrate the safety of switching from DAPT to aspirin monotherapy 3 months after ZES implantation in patients with CAD.

The optimal duration of DAPT after DES has not been fully established. The PCI-CURE [19], CHARISMA [20], CREDO [21], and other clinical studies have shown that extended oral administration of clopidogrel after DES implantation can reduce the risk of cardiovascular complications, for example, cardiac death and MI. Other clinical studies [4, 7, 22] have shown no obvious clinical benefits of DAPT lasting for 6 months or more. The CHARISMA study, the only randomized study among the above studies, did not demonstrate the clinical benefits of continued DAPT for asymptomatic patients. There has been no randomized prospective study that has truly demonstrated clinical benefits of the long-term continuation of DAPT.

DAPT can be a risk factor for bleeding events. In the CHARISMA study, the incidence of major bleeding causing hemodynamic disruption in the 2-year continued DAPT group was 1.7% [20]. What should be noted is the fact that the incidence of major bleeding is higher than the incidence of ST that may develop during the same time span. Asian people are considered to be at high risk for intracranial hemorrhage [23]. A prospective multicenter observational study in Japanese patients undergoing continued DAPT has shown an annual incidence of 0.6% for intracranial hemorrhage and an overall annual incidence of 16.6% for bleeding events [24]. Therefore, DAPT is considered to be an independent risk factor for bleeding events. On the other hand, 5-year followup after PCI showed that 26% of patients underwent noncardiac surgery [25]. In another study, 6.3% of patients underwent noncardiac surgery requiring the discontinuation of DAPT within 1 year after ZES implantation [17]. If we could switch DAPT to aspirin monotherapy early after DES implantation, the risk of bleeding events could be lowered and the problems associated with surgery could be lessened, resulting in improvement in the quality of life of patients. Previous studies have provided valuable evidence regarding the incidences of bleeding events associated with DAPT, but there has been limited information on the safety and clinical outcomes of early switching from DAPT to aspirin monotherapy after DES implantation that is required by cardiologists. A prospective study to assess the safety of discontinuing clopidogrel in a short time span after ZES implantation is ongoing [26, 27]. We believe that a shorter duration of DAPT after ZES should be associated with better quality of life of patients undergoing PCI. 

Among DESs, ZES has good product profiles for a short duration of DAPT. ZES rapidly releases an antiproliferative drug to surrounding tissue, enhancing the coverage of endothelial cells on it. A study by optical coherence tomography (OCT) showed complete neointimal coverage as early as 3 months after ZES implantation [28]. Another study by OCT showed that ZES has few bare-stent struts and little thrombosis compared to a sirolimus-eluting stent at 9 months after implantation [29]. ZES showed significantly less acetylcholine-induced vasoconstriction than did a sirolimus-eluting stent [15]. These studies indicate high safety profiles of ZES: acceleration of reendothelialization and preservation of endothelial function. In the present study, therefore, we used ZES as a DES with the greatest potential for shortening the duration of DAPT for the reasons discussed above.

Early switching from DAPT to aspirin monotherapy after DES implantation may increase the risk of ST. Hahn et al. reported that discontinuation of DAPT at 3 month after ZES implantation was not associated with an increased incidence of ST [27]. None of the observational studies on the relationship between DAPT duration and ST succeeded in showing a relationship between late ST and DAPT duration. In this study, we showed that DAPT for 3 months after ZES implantation is not associated with risk of ST compared with DAPT for 12 months. On the other hand, the short duration of DAPT may increase the risk of in-stent restenosis because platelets that have adhered to the stent may release chemoattractants and proliferative factors to enhance the migration and proliferation of vascular smooth muscle cells. However, there was no difference in MLD, late loss, and %DS between the two groups at 8 months after implantation. There was also no difference in the incidence of MACE between the two groups. Consequently, our study has provided useful information for the shortening of the duration of DAPT in the real-world clinical setting.

The present study has several limitations. First, the study was retrospectively conducted at a single medical institution and the study population was small. Second, the patients in the 3-month DAPT group were not selected randomly, and selection bias may exist in the preventive effect of DAPT on cardiovascular events and in baseline characteristics of patients. Third, the number of ST was very low in both groups. Longer observation will be needed to evaluate the incidence of ST on very late phase. Fourth, the study used ZES only and is therefore not a controlled study using another DES. Since the assessment of safety and efficacy may differ depending on the type of DES, further study will be needed to corroborate the clinical evidence obtained in the present study.

## 5. Conclusion

The 3-month DAPT group was equivalent to the 12-month DAPT group in clinical outcomes and overall incidence of MACE. The incidence of bleeding events was significantly lower in the 3-month DAPT group than in the 12-month DAPT group. Therefore, the present study suggests that DAPT can be safely discontinued at 3 months after ZES implantation in patients with CAD.

Although there was no difference in the incidence of ST between the two groups, the 12-month DAPT group had longer lesion length. The short duration of DAPT therapy may apply to longer stent length.

## Figures and Tables

**Table 1:** Baseline characteristics of patients in the 3- and 12-month DAPT groups.

	3-month DAPT *n* = 67	12-month DAPT *n* = 61	*P* value
Male gender, *n* (%)	43 (64.8)	31 (51.8)	0.05
Age, yrs	70.3 ± 9.8	70.8 ± 9.2	NS
Stable angina, *n* (%)	52 (78.2)	48 (78.7)	NS
Acute coronary syndrome, *n* (%)	15 (21.8)	14 (23.0)	NS
Prior myocardial infarction, *n* (%)	10 (14.8)	8 (13.1)	NS
Prior PCI, *n* (%)	18 (27.3)	18 (29.5)	NS
Prior CABG, *n* (%)	3 (4.5)	1 (1.6)	NS
Heart failure, *n* (%)	7 (10.2)	8 (13.1)	NS
Diabetes mellitus, *n* (%)	33 (49.2)	30 (49.2)	NS
Hypertension, *n* (%)	59 (87.5)	49 (80.3)	NS
Dyslipidemia, *n* (%)	50 (74.6)	46 (75.4)	NS
Current smoker, *n* (%)	34 (50.8)	21 (34.3)	0.02
Cr: >2.0 mg/dL, *n* (%)	5 (6.8)	7 (11.5)	NS

DAPT: dual antiplatelet therapy; PCI: percutaneous coronary intervention; Cr: creatinine; NS: not significant.

**Table 2:** Lesion characteristics and coronary angiographic outcomes at baseline, immediately after and at 8 months after implantation of the endeavor zotarolimus-eluting stent in the 3- and 12-month DAPT groups.

	3-month DAPT (88 lesions)	12-month DAPT (81 lesions)	*P*-value
Baseline			
RCA, *n* (%)	36 (40.5)	28 (34.6)	
LAD, *n* (%)	33 (37.5)	29 (35.8)	NS
LCX, *n* (%)	15 (17.1)	18 (22.2)	
LMT, *n* (%)	4 (4.9)	6 (7.4)	NS
AHA/ACC Type B1, *n* (%)	22 (24.7)	26 (32.0)	
Type B2, *n* (%)	54 (61.6)	43 (53.0)	NS
Type C, *n* (%)	5 (5.7)	3 (3.7)	
* De novo, n (%) *	86 (97.7)	79 (97.5)	NS
In-stent restenosis, *n* (%)	2 (2.3)	2 (2.5)	NS
Bifurcation lesion, *n* (%)	6 (6.8)	7 (8.6)	NS
Multivessel disease, *n* (%)	18 (20.5)	20 (24.7)	NS
Lesion length, mm	13.3 ± 7.04	15.6 ± 5.82	0.02
RVD, mm	2.78 ± 0.52	2.74 ± 0.53	NS
MLD, mm	0.69 ± 0.43	0.71 ± 0.39	NS
%DS, %	73.9 ± 17.3	73.2 ± 14.9	NS
Immediately afterprocedure			
Multiple stenting, *n* (%)	19 (21.5)	15 (18.5)	NS
Number of stents per patient	1.7 ± 1.1	1.8 ± 0.7	NS
MLD, mm	2.39 ± 0.56	2.51 ± 0.61	NS
%DS, %	9.07 ± 7.56	7.94 ± 6.69	NS
8 months afterprocedure			
RVD, mm	2.38 ± 0.53	2.64 ± 0.51	NS
MLD, mm	2.24 ± 0.58	2.16 ± 0.79	NS
%DS, %	19.7 ± 17.3	21.4 ± 20.2	NS
Late lumen loss, mm	0.45 ± 0.52	0.51 ± 0.47	NS
Binary restenosis, *n* (%)	8 (9.1)	9 (11.1)	NS

RCA: right coronary artery; LAD: left coronary artery; LCX: left circumflex artery; RVD: reference vessel diameter; MLD: minimal lumen diameter; TLR: target lesion revascularization; TVR: target vessel revascularization; LMT: left main trunk; DAPT: dual antiplatelet therapy; DS: diameter stenosis; NS: not significant.

**Table 3:** Incidences of sent thrombosis in the 3- and 12-month DAPT groups after implantation of the endeavor zotarolimus-eluting stent.

	3-month DAPT (88 lesions)	12-month DAPT (81 lesions)	*P*-value
Stent thrombosis, *n* (%)	0 (0)	1 (1.2)	NS
0–30 days, *n* (%)	0 (0)	0 (0)	NS
31–365 days, *n* (%)	0 (0)	1 (1.2)	NS

DAPT: dual antiplatelet therapy; NS: not significant.

**Table 4:** Incidences of major adverse cardiac events in the 3- and 12-month DAPT groups.

	3-month DAPT (88 lesions)	12-month DAPT (81 lesions)	*P*-value
MACE, *n* (%)	10 (11.4)	9 (11.1)	NS
Death (all causes), *n* (%)	0 (0)	1 (1.2)	NS
Cardiac death, *n* (%)	0 (0)	1 (1.2)	NS
MI (all causes), *n* (%)	2 (2.3)	1 (1.2)	NS
Q-MI, *n* (%)	1 (1.1)	1 (1.2)	NS
Non-Q-MI, *n* (%)	1 (1.1)	0 (0)	NS
Cerebrovascular accident, *n* (%)	0 (0)	1 (1.2)	NS
TLR, *n* (%)	8 (9.1)	8 (9.9)	NS
TVR, *n* (%)	8 (9.1)	8 (9.9)	NS

DAPT: dual antiplatelet therapy; MACE: major adverse cardiac events; MI: myocardial infarction; TLR: target lesion revascularization; TVR: target vessel revascularization; NS: not significant.
